# Forest structural changes affect dung beetle communities and dung removal rates

**DOI:** 10.1007/s00442-026-05974-7

**Published:** 2026-10-09

**Authors:** Julian Lunow, Katja Wehner, Matteo Trevisan, Michael Heethoff, Nico Blüthgen, Nadja K. Simons

**Affiliations:** 1 Biocenter, Chair of Conservation Biology and Forest Ecology, Applied Biodiversity Science, Julius-Maximilians-University Würzburg, 97074 Würzburg, Germany; 2 Institute of Ecology, Leuphana University Lüneburg, 21335 Lüneburg, Germany; 3 Ecological Networks, Technical University Darmstadt, 64287 Darmstadt, Germany; 4 Animal Evolutionary Ecology, Technical University Darmstadt, 64287 Darmstadt, Germany

**Keywords:** Dung decomposition, Forest heterogeneity, Geotrupidae, Scarabaeidae, Tree dieback

## Abstract

Forest ecosystems are shaped by the consequences of management activities, as well as through an increase in tree damage and mortality caused by climate change. These drivers lead to alterations in forest structure, affecting the abundance, diversity, and composition of organisms, as well as their provision of ecosystem functions. However, relatively little is known about how dung beetles, which are important for ecosystem functioning, respond to recent changes in forest characteristics. We monitored dung beetles and dung decomposition in 67 forest plots in Darmstadt, Germany. The structure of these plots varied considerably due to tree mortality caused by several years of drought and heatwaves, as well as former forest management practices in the study region. The total biomass, diversity, and species composition of dung beetles strongly corresponded to changes in forest structure, such as mean canopy openness, mean tree diameter at breast height, and forest ground cover. However, they were less related to within-plot environmental heterogeneity. Higher dung beetle biomass was related to a higher dung decomposition rate, but changes in dung beetle diversity and community composition did not correlate with dung decomposition. Our results suggest that changes in forest structure result in changes in the biomass, diversity, and composition of dung beetle communities. The relationships between dung beetles and structural heterogeneity were not particularly pronounced. This indicates that the effects of forest management and tree mortality on dung beetles and subsequent changes in dung decomposition are mediated by directed shifts in mean forest structure rather than structural heterogeneity.

**Supplementary Information:**

The online version contains supplementary material available at https://doi.org/10.1007/s00442-026-05974-7.

## Introduction

Forests cover a significant proportion of Europe’s land area, and the proportion of forests designated primarily for biodiversity conservation is increasing (FAO 2020). This is promising for biodiversity and ecosystem functioning in forest ecosystems. Following the niche theory, one key factor in promoting the diversity of organisms in forest ecosystems is increasing structural heterogeneity, with subsequent effects on ecosystem functioning (Chesson 2000; Eisenhauer et al. 2023; Turnbull et al. 2016). However, the structure of forests has been shaped by forest management for centuries, particularly due to the increased intensity of management over the last 200 years (McGrath et al. 2015; Pan et al. 2013). This includes factors such as the availability of deadwood, introducing non-native tree species and the heterogeneity of tree sizes (Asplund et al. 2024). Small-scale changes in forest structures resulting from forest management affect the species richness of different taxonomic groups or functional groups (Chaudhary et al. 2016; Lelli et al. 2019; Spake et al. 2015), with differences observed on regional scales (Schall et al. 2018). Furthermore, forests are currently experiencing the effects of heat waves and drought (Allen et al. 2010; Senf et al. 2020). Severe weather events, which are caused by human-induced climate change, pose physiological challenges for trees and can result in tree dieback (Albrich et al. 2020; Menezes-Silva et al. 2019; Schuldt et al. 2020). Large-scale changes, such as forest habitat degradation and fragmentation affect biodiversity (Brockerhoff et al. 2017; Newbold et al. 2015; Seibold et al. 2019). Additionally, tree dieback has been shown to alter community composition and the diversity of different animal taxa, as well as plant cover (Cours et al. 2022; Kirby et al. 2022; Sire et al. 2022). Overall, forest management and tree dieback directly impact both general environmental site conditions and small-scale structural heterogeneity, subsequently affecting biodiversity (Heidrich et al. 2020; Leidinger et al. 2021; Staab et al. 2023). As environmental change continues to alter forest structural features, understanding their differential effects on different taxa remains a key research objective. Furthermore, the provision of ecosystem services and functions is under threat, which could lead to economic and environmental crises (Felipe-Lucia et al. 2018; Malhi et al. 2020; Millar and Stephenson 2015), posing challenges to ecosystems and humanity (Cardinale et al. 2012; Sala et al. 2000). Given the importance of arthropods in terms of biomass and diversity for the functioning of ecosystems (Buse and Entling 2020; Lefcheck et al. 2015), it is crucial to identify the habitat characteristics that support arthropod communities with specific roles in ecosystem functioning in temperate forests.

In this study, we explicitly distinguish between average forest structure in a plot (e.g. mean tree diameter at breast height (DBH) of trees on a plot) and small-scale heterogeneity of forests structures within a plot (e.g. variability in mean DBH of trees in a plot). The diversity of trees or other resources for organisms can also be viewed as a measure of heterogeneity. In general, forest structure and heterogeneity are key ecological features that influence the diversity and composition of arthropod communities. Both, mean conditions (mirrored by an average structure) as well as heterogeneity of the conditions (variation in structures, or diversity of resources) are known to shape the abundance, diversity and community composition of different taxa (Knuff et al. 2020; Larrieu et al. 2019; Penone et al. 2019). Important aspects of forest structure that are related to arthropods are mean canopy opening, microclimate, deadwood volume, tree size and tree identity (Doerfler et al. 2018; Penone et al. 2019; Staab et al. 2023). But depending on their ecological characteristics and habitat preferences, arthropod communities react positive or negative to a warmer and drier microclimate due to higher canopy opening and changes in the microclimate (Gandhi et al. 2008; Lettenmaier et al. 2022; Perry et al. 2018; Staab et al. 2022). Whereas tree size had no consistent effect on beetle communities (positive: (Knuff et al. 2020), neutral: (Penone et al. 2019), an increase in deadwood volume has consistently positive effects on the abundance and diversity of beetles (Doerfler et al. 2018; Larrieu et al. 2019; Penone et al. 2019). Furthermore, the proportion of non-native trees plays a crucial role in shaping the ecological dynamics of forest arthropod communities, particularly in terms of diversity, community composition and temporal changes (Penone et al. 2019; Staab et al. 2023).

The effects of forest structural heterogeneity on arthropod communities, including vertical heterogeneity, deadwood diversity and tree diversity, have been investigated in fewer studies, with inconsistent results (Heidrich et al. 2020; Knuff et al. 2020; Larrieu et al. 2019; Staab et al. 2023). Increasing deadwood diversity positively affects saproxylic beetle diversity (Chamagne et al. 2016; Heidrich et al. 2020), while higher tree diversity positively influences insect abundance trends over time (Staab et al. 2023). Much is known about insects that decompose deadwood, but little is known about dung-decomposing beetles’ reactions to changes in forest structures and structural heterogeneity.

Dung beetles (Coleoptera: Geotrupidae; Scarabaeidae) play an important role in ecosystem functioning because they can improve soil quality and nutrient cycling, thereby increasing plant growth (Anderson et al. 2024; Nichols et al. 2008; Slade et al. 2016). In a full-factorial experiment, Staab et al. (2022) found differences in dung beetle diversity, biomass, and dung removal rates between forest gaps and closed forests. This demonstrates that changes in structural features, such as the creation of forest gaps following total tree mortality or clear-cutting, are associated with a decrease in dung beetle biomass and, consequently, their functional role in ecosystems. The key function of dung removal is mainly influenced by habitat type and dung beetle biomass (Buse and Entling 2020). Numerous studies have demonstrated that the species *Anoplotrupes stercorosus* accounts for the greatest biomass and plays the pivotal role in dung decomposition in temperate European forests (Buse and Entling 2020; Hülsmann et al. 2020; Staab et al. 2022). As *A. stercorosus* seems to act as a keystone species for dung decomposition and therefore ecosystem functioning in forest ecosystems, it is crucial to analyse responses of this species, as well as those with similar morphological traits, to changes in mean forest structure and structural heterogeneity. This is particularly pertinent in forests where the structure and small-scale heterogeneity are currently changing due to forest management and increasing tree mortality. It is important to understand how changes in forest structural characteristics influence dung beetle communities and their relevant contribution to ecosystem functioning.

Our study examined how variation in average environmental conditions and forest structure per plot affect dung beetle communities and the removal of dung. Structural variation at least partly results from past forest management practices and the current dieback of trees. To this end, we aimed to answer the following research questions:

1. Do dung beetle biomass, diversity and community composition respond to the substantial variation in forest structures?
2. Do effects correspond to average site conditions (mean structural parameters) or to their small-scale heterogeneity (variability of the structural parameters, tree diversity), or both?
3. Do changes in the dung beetle community translate to altered dung decomposition rates?

## Methods

### Study site and forest characteristics

The study was carried out in two different regions (East and West orientation) of the municipal forest of Darmstadt (Hesse, Germany). The forests in the study area remained unmanaged since 2019. The soil conditions differ between the regions, with nutrient-poor and water-deficient sandy soils dominating in the western part and water-retaining loamy soils in the eastern part (Wehner et al. in press). The forest plots in the study area are characterised by different native and non-natural main tree species. The native main tree species are beech (*Fagus sylvatica*), oak (*Quercus petraea/robur*) and black alder (*Alnus glutinosa*). In addition, planted pine (*Pinus sylvestris*), planted Norway spruce (*Picea abies*) and self-seeding mountain black cherry trees (*Prunus serotina*) dominate other plots. In the Darmstadt municipal forest, 320 inventory points (250 m apart) are inventoried annually according to the guidelines of the German National Forest Inventory (Meining 2024). We established study plots of 30 × 30 m around 67 of those inventory points (Figure S1). On the plots we identified all living trees (> 7 cm in diameter, as 5–7 cm diameter thresholds are most commonly used in European wide forest inventories (Burrascano et al. 2021) and measured their diameter at breast height (DBH) (~ 1.3 m). These data were used to calculate mean tree DBH, coefficient of variation (CV) of tree DBH and total tree basal area ($\sum_{i=1}^{n}\pi\:* \left(\frac{{DBH}_{i}}{2}\right)^{2},$ in cm²) per plot. Mean and CV of tree height per plot were estimated using stand height curves derived from measurements of selected trees per plot and tree species. Individual tree heights were measured using a laser distance meter (Bosch Universal Distance 40 C). For each plot we also calculated tree species richness and tree diversity (e^H^; Jost 2006) based on the basal area of each tree species per plot. The proportion of non-natural trees (*Pinus strobus*,* Pinus sylvestris*,* Picea abies*,* Larix europeana*,* Prunus serotina*,* Quercus rubra*,* Robinia pseudoacacia*,* Alianthus altissima*,* Aesculus hippocastanum*) was computed by dividing the sum of the basal area of the non-natural tree species by the overall tree basal area. We considered Scots pine (*Pinus sylvestris*) and European spruce (*Picea abies*) as non-natural tree species because they would be very rare (Scots pine) or absent (European spruce) in the study region without human introduction (Suck et al. 2014). To calculate the volume of deadwood, we inventoried all standing dead trees on the plots with a DBH > 7 cm by measuring DBH and height. For stumps and lying deadwood (> 25 cm in diameter) all pieces were measured by three diameters (beginning, mid, ending or top, middle, bottom) and height/length. Volume of smaller dead stumps and lying deadwood (7–25 cm in diameter) was estimated using both diagonal lines from one corner of the plot to the other (~ 42 m) following the line-intersect method of van Wagner (1982). Canopy openness was assessed by densiometer measurements (Forestry Suppliers, Inc, Jackson, Mississippi, USA.) at the SW corner, NE corner and centre of each plot. At each measurement point, three different people measured canopy openness for each of the four compass directions. Canopy openness per plot was then calculated as the mean of these 36 measurements. We also calculated the CV of canopy openness based on the 36 measurements. We used estimates of stand age and visible tree damage in the crown [%] (hereafter defoliation) for the eight biggest trees around the plot midpoint, as reported in the annual forest inventory report (Meining 2024). Defoliation values were estimated in 5% increments by visual inspection of the tree crown. Forest ground cover was inventoried in five 2 × 2 m squares, oriented on a diagonal line from the southeast to the northwest corner of the plots and approximately 10 m apart. In these squares we estimated the cover of moss, foliage, herbs, deadwood, small and large trees and bare ground and measured the height of the foliage. In order to obtain a value for all forest ground parameters per plot, we calculated a mean value for each cover parameter from the five squares per plot. Based on the mean cover values for each plot, we also calculated the evenness of the ground cover. All measurements of forest characteristics were taken in 2023 and 2024.

### Dung beetle and dung decomposition survey

Dung beetles were sampled in two surveys in June 2023 and June 2024 using baited pitfall traps. We placed one baited pitfall trap on each of the 67 plots. We used water with a drop of soap as the trapping fluid in these traps. The traps, with an opening of 9 cm in diameter and a volume of 400 ml, were filled with 250 ml of the trapping liquid. We used tea bags filled with cow dung as bait. The dung was filled into tea bags (~ 30 g) and each tea bag was placed ~ 5 cm above the trap by attaching the bag to a wooden stick with a rubber band. Prior to deployment, the filled tea bags were frozen at − 18 °C and removed approximately one hour before deployment. The traps were set out over two days. All dung beetles sampled were preserved in 70% ethanol and identified to species level.

The protocols of Frank et al. (2017) and Staab et al. (2022) were used for the dung decomposition survey on all 67 plots in 2024. We weighed 190 g of fresh cow dung (mean 189.07 ± 6.37 g SD) in separate plastic bags. These bags were frozen at − 18 °C. On the plots, the cow dung from one plastic bag was placed on cellulose paper. After 48 h, the dung was collected and the attached debris was carefully removed. The dung was then oven-dried (60 °C) and reweighed. To calculate the pre-exposure dry weight, we dried four additional dung samples and calculated their mean weight loss. This mean weight loss was used as a conversion factor from fresh to dry dung weight. Dung removal was calculated as the difference between the pre-exposure dry weight and the post-exposure dry weight divided by the pre-exposure dry weight. Removal was only calculated for samples with holes in the cellulose paper to verify that missing dung could be associated to dung beetle activity (Frank et al. 2017; Staab et al. 2022). If no holes were detected, dung removal was set to 0 but included in the analysis. Cow dung for the traps and the dung decomposition survey was provided by an organic farm (Hofgut Oberfeld, Darmstadt, Germany) where no vermicides are used and the cows are fed only grass and hay.

### Statistical analysis

**Fig. 1:**
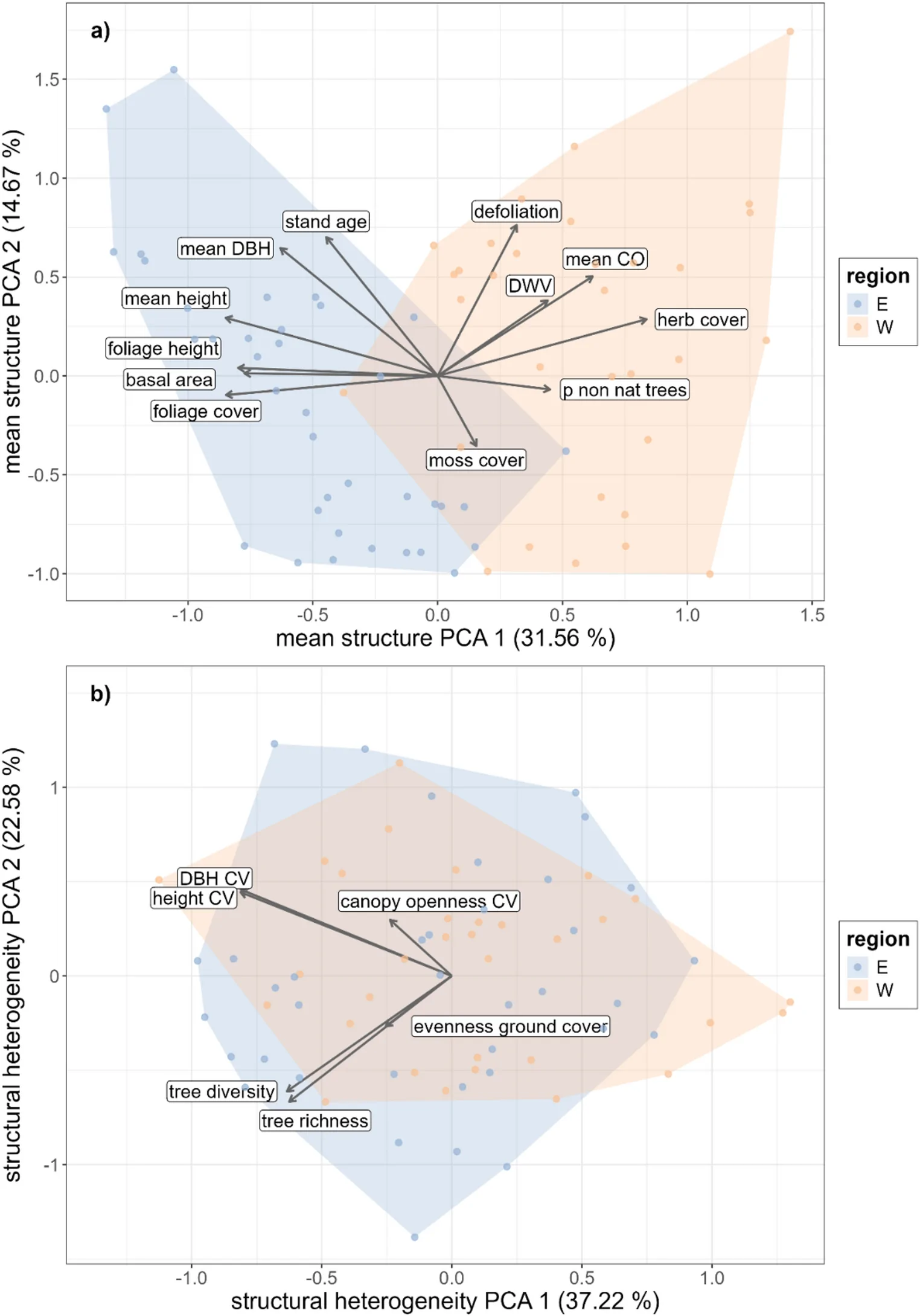
Principal component analysis (PCA) ordination of the studied plots for mean forest structure (**a**) and structural heterogeneity (**b**). The colours represent the two different regions (E = East, W = West) in the municipal forest of Darmstadt, DE. The variables included in each of the PCAs can be found in Table S1. In the mean forest structure PCA only significant variables are depicted. In the structural heterogeneity ordination, all included variables were significant and depicted. Full details on the variables can be found in Table S1. Percentage in the axis titles values represent the explained variance of each PCA axis. With the abbreviation “DBH” is “tree diameter at breast height”, “DWV” is “deadwood volume”, “CO” is “canopy openness”, “p non nat trees” is “proportion of non-natural trees” and “gc” is “ground cover”

Analyses were performed using R 4.3.1 (R Core Team 2023). We computed two principal component analyses (PCAs): one PCA for the 16 mean structural parameters (Fig. 1a, Table S1), and one PCA for the 6 small-scale structural heterogeneity parameters (e.g. the spatial CV of DBH in a plot) (Fig. 1b, Table S1). Before calculating the PCA, each parameter was scaled (mean = 0, SD = 1). We extracted the first two axes for each PCA for further analysis. The two PCA axes for the mean forest structure explained 46.23% of the variance (axis 1: 31.56%, axis 2: 14.67%) and the two PCA axes for the structural heterogeneity explained 59.8% (axis 1: 37.22%, axis 2: 22.58%). We assessed the significance and association with the PCA axes of the variables fitted to the PCA ordination space using a post-hoc permutation test (999 permutations) (Table S1).

The dry biomass in g for each dung beetle species was derived from mean species specific biomass calculations of Frank et al. (2017). The total dry biomass of dung beetles [g] per plot was calculated by summing the products of the mean species-specific biomass and the number of individuals of each species in each plot. The diversity of the dung beetle community was calculated as species richness and the exponential Shannon diversity (e^H^; Jost 2006). To test for differences in community composition, we calculated Bray-Curtis distance matrices on the basis of proportional abundance and on the basis of proportional biomass values (sqrt-transformed) per plot. Additionally, we calculated Jaccard distance matrices to analyse community compositional changes without weighting for species abundance or biomass. Species abundances for *Anoplotrupes stercorosus*, *Trypocopris vernalis* and *Volinus sticticus* were converted to presence-absence data due to their occurrence on some, but not all plots, which limited the applicability of abundance-based models. We decided to keep *A. stercorosus* and *T. vernalis* in the analysis because large tunneling dung beetle species are the most effective group for dung removal in Central Europe (Buse and Entling 2020; Milotić et al. 2019). In addition, we kept *V. sticticus* as a small dung-dwelling and very abundant species in temperate European forests (Buse et al. 2018).

There were only moderate correlations (Pearson’s *r* < 0.4, Figure S2) between the four different PCA axes, none of which were significant based on the *cor.mtest* function of the *corrplot* package (Wei et al. 2017; Table S2). To account for the effects of the PCA axes on biomass and diversity per plot, we modelled their interrelationship using linear models with biomass (sqrt-transformed), species richness or Shannon diversity (e^H^) as response, mean forest structure and structural heterogeneity PCA axes 1 and 2 as explanatory variables, and year as a random factor. To analyse the influence of the four PCA axes on the dung beetle community ordinations, we correlated the scores of the first two non-metric multidimensional scaling (NMDS) axes with the four environmental PCA axes in a post-hoc permutation test (999 permutations) using the *vegan* package (Oksanen et al. 2022). We used generalized linear mixed models (*glmmTMB* function from *glmmTMB* package (Brooks et al. 2017) with a binomial distribution and year as a random factor to test the effects of the four environmental PCA axes on the presence/absence of *A. stercorosus*,* T. vernalis* and *V. sticticus* on the plots, to account for changes in their occurrence due to changes in forest structural characteristics. The effects of dung beetles and the environment on dung removal were analysed using linear models with dung beetle biomass (overall, *A. stercorosus* or *T. vernalis* biomass; sqrt-transformed), species richness, Shannon diversity (e^H^) and the four PCA axes as explanatory variables. The correlation between the three dung beetle community ordinations and dung removal was tested using a post-hoc permutation test (999 permutations) with the *vegan* package (Oksanen et al. 2022). As dung removal was only assessed in 2024, we only used the dung beetle community data from 2024 in this analysis. The analyses including both years were performed on 133 data points (67 plots in 2023 and 66 plots in 2024), but the analyses of dung removal in 2024 were only performed on 66 data points due to a trap failure in one plot. All model residuals were checked for normal distribution, heteroscedasticity and the models themselves for overdispersion. Model assumptions were always met after transforming some of the variables. Furthermore, we tested if the raw data differs between the East (loamy soil conditions) and West (sandy soil conditions) region using two-tailed t-tests and found that it is likely to be spatially autocorrelated (Figure S3-S4). Therefore, it was not possible to include region as a fixed effect in addition to the environmental variables, as this would severely inflate variances. In line with that, the model residuals were checked for spatial autocorrelation post hoc, to assess whether environmental effects could be confounded by the spatial clustering of other environmental condition (e.g. soil properties) between eastern and western regions (Dormann et al. 2007). We used Moran’s I test within the *testSpatialAutocorrelation* function of the *DHARMa* package (Hartig 2024). For this, the dataset was subdivided into single datasets for 2023 and 2024, models of the same structure for the two years were fitted and the model residuals were entered into the function. Most model residuals were not spatially autocorrelated. However, the model for species richness was spatially autocorrelated, a pattern that persisted even after the region was included as a random effect. In contrast, after including the region as a random factor in the *A. stercorosus* occurrence model spatial autocorrelation no longer occurred (Figure S5-S6; Table S3). We further explored potential differences in model residuals between the two study regions using ANOVA or Wilcoxon-test with scaled model residuals as response and region as explanatory variable (Table S4). Only the scaled model residuals for the *A. stercorosus* model without inclusion of region (F_1;65_ = 5.695, *p* = 0.02) and the *V. sticticus* model (F_1;65_ = 4.689, *p* = 0.034) showed differences between regions, with higher values in the eastern study region in 2023 (Table S4).

## Results

**Fig. 2:**
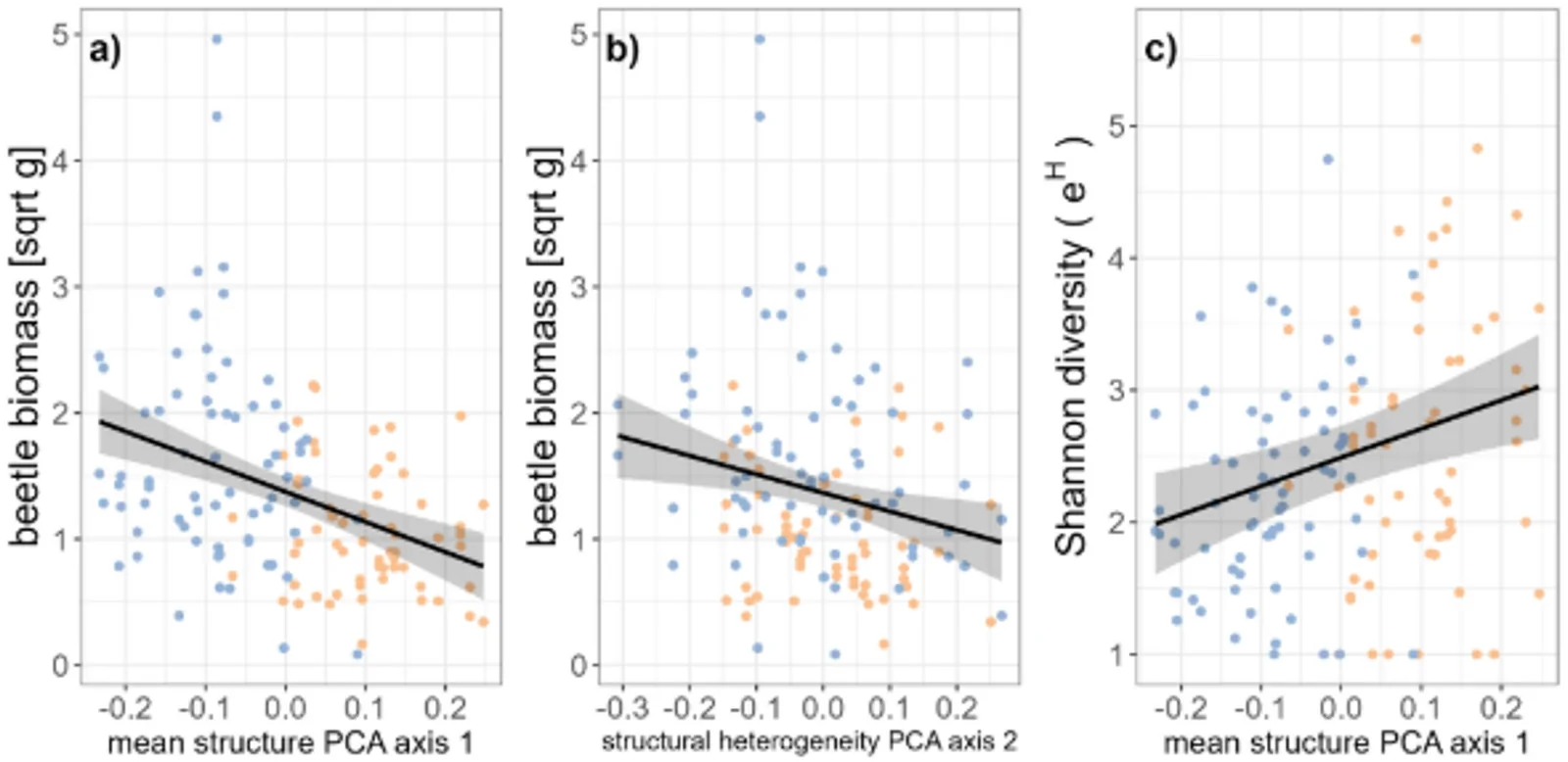
Effects of mean forest structure and structural heterogeneity PCA axes on total dry dung beetle biomass [g] (sqrt-transformed) and Shannon diversity (e^H^). (**a**) Effect of mean forest structure PCA axis 1 on dung beetle biomass. (**b**) Effect of structural heterogeneity PCA axis 2 on dry dung beetle biomass [g] (sqrt-transformed). (**c**) Effect of mean forest structure PCA axis 1 on dung beetle Shannon diversity. The coloured points represent the two different study areas (blue = eastern forest, orange = western forest). The full model results are given in Table S6

**Fig. 3:**
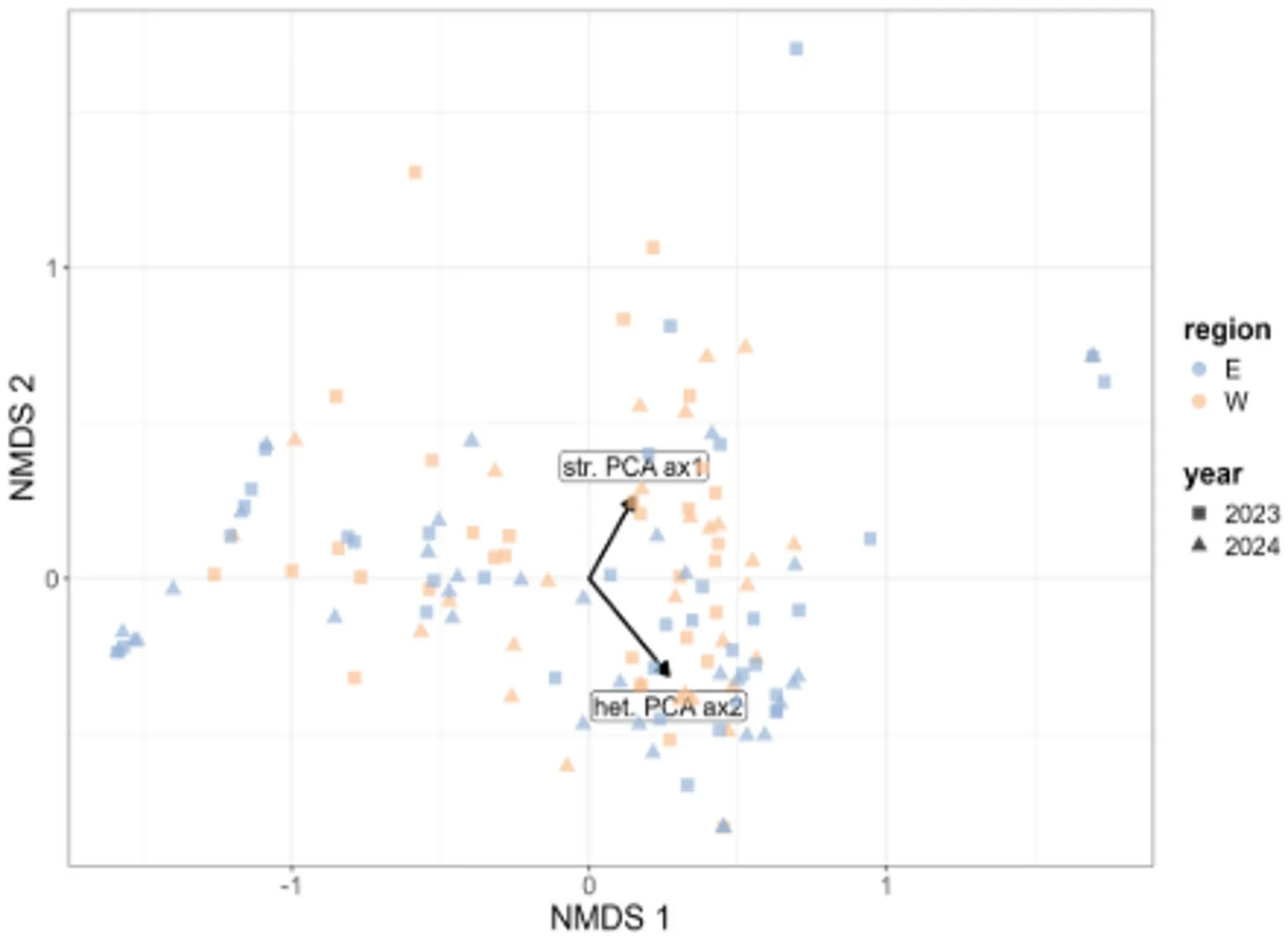
NMDS on biomass-based dung beetle community composition (stress = 0.093). The colours represent the two different study regions, the rectangles communities sampled in 2023 and the triangles communities sampled in 2024. The arrows depict significant effects in the PERMANOVA of mean forest structure PCA axis 1 (str. PCA ax1) and structural heterogeneity PCA axis 2 (het. PCA ax2) on the community composition to only indicate that there are significant influences of the variables on community composition. The full PERMANOVA results are given in Table S7

In both years together, we sampled 10,743 dung beetle individuals of 15 species (3 Geotrupidae, 12 Scarabaeidae) with a total calculated dry biomass of 370.91 g. The most abundant species was the small scarabeid *Volinus sticticus* (62.48% of all individuals), and the large geotrupid *Anoplotrupes stercorosus* contributed 70.75% of the total dry biomass (Table S5). However, the contribution of *A. stercorosus* to dung beetle biomass per plot varied considerably (mean = 0.517, SD = 0.363). Total dry biomass of dung beetles was negatively related to mean forest structure PCA axis 1, i.e. dung beetle biomass decreased from native forests with taller trees, a larger tree basal area and a higher canopy cover to forests with lower trees, a higher proportion of non-natural trees and a higher ground cover of herbs (Fig. 2, Table S6). Total dung beetle biomass also responded to changes in forest structural heterogeneity (forest structural heterogeneity PCA axis 2), because decreases in total dung beetle biomass were associated with a less diverse tree community, more heterogeneous ground cover and greater canopy diversification (Fig. 2, Table S6). In contrast to the total dry biomass, Shannon diversity of dung beetles increased from native forests with taller trees, a larger tree basal area and a higher canopy cover to forests characterised by lower tree height, a higher proportion of non-natural trees and a higher ground cover of herbs (mean forest structure PCA axis 1; Fig. 2), while species richness did not respond to forest structural characteristics (Table S6). Changes in the dung beetle community, calculated on the basis of biomass, were associated with the aforementioned average conditions (mean forest structure PCA axis 1) and small-scale structural heterogeneity parameters (forest structural heterogeneity PCA axis 2), but not unweighted species composition or based on abundance (Fig. 3, Table S7).

**Fig. 4:**
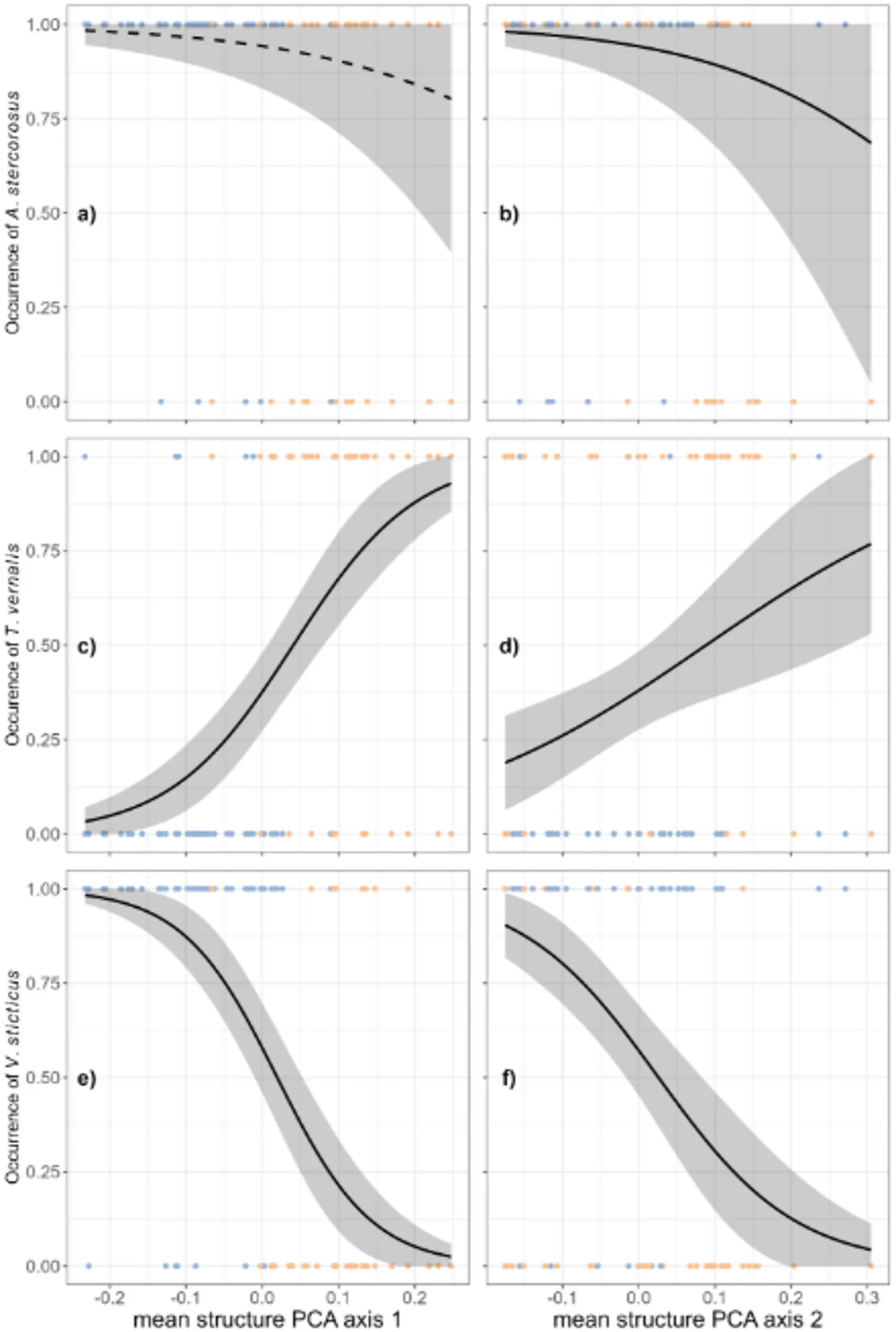
Effects of mean forest structure PCA axis 1 (**a**,** c**,** e**) and mean forest structure PCA axis 2 (**b**,** d**,** f**) on the occurrence of *A. stercorosus* (**a**,** b**; 109 plots with, 24 without occurence), *T. vernalis* (**c**,** d**; 54 plots with, 79 without occurence) and *V. sticticus* (**e**,** f**; 76 plots with, 57 without occurence). The solid lines represent significant effects and the dashed line marginal significant effects. Light grey colours indicate the 95% confidence intervals. The coloured points represent the two different study areas (blue = eastern forest, orange = western forest). The full model results are given in Table S8

The two most abundant species (*A. stercorosus*, *V. sticticus*) and *Trypocopris vernalis* as the functionally almost similar species to *A. stercorosus* responded to changes in mean forest structure, but not to structural heterogeneity. The probability of occurrence of *A. stercorosus* and *V. sticticus* was high in forests with larger tree basal area, more native trees, taller trees, and higher canopy cover, and decreased in forests characterised by opposite structural conditions (mean forest structure PCA axis 1; Fig. 4a, e, Table S8). Furthermore, the occurrence of *A. stercorosus* and *V. sticticus* was negatively associated with increasing tree defoliation and mean canopy openness (mean forest structure PCA axis 2; Fig. 4b, f, Table S8). The responses of *T. vernalis* were contrasting to responses of *A. stercorosus* and *V. sticticus* along both mean forest structure PCA axes (Fig. 4c, d, Table S8).

**Fig. 5:**
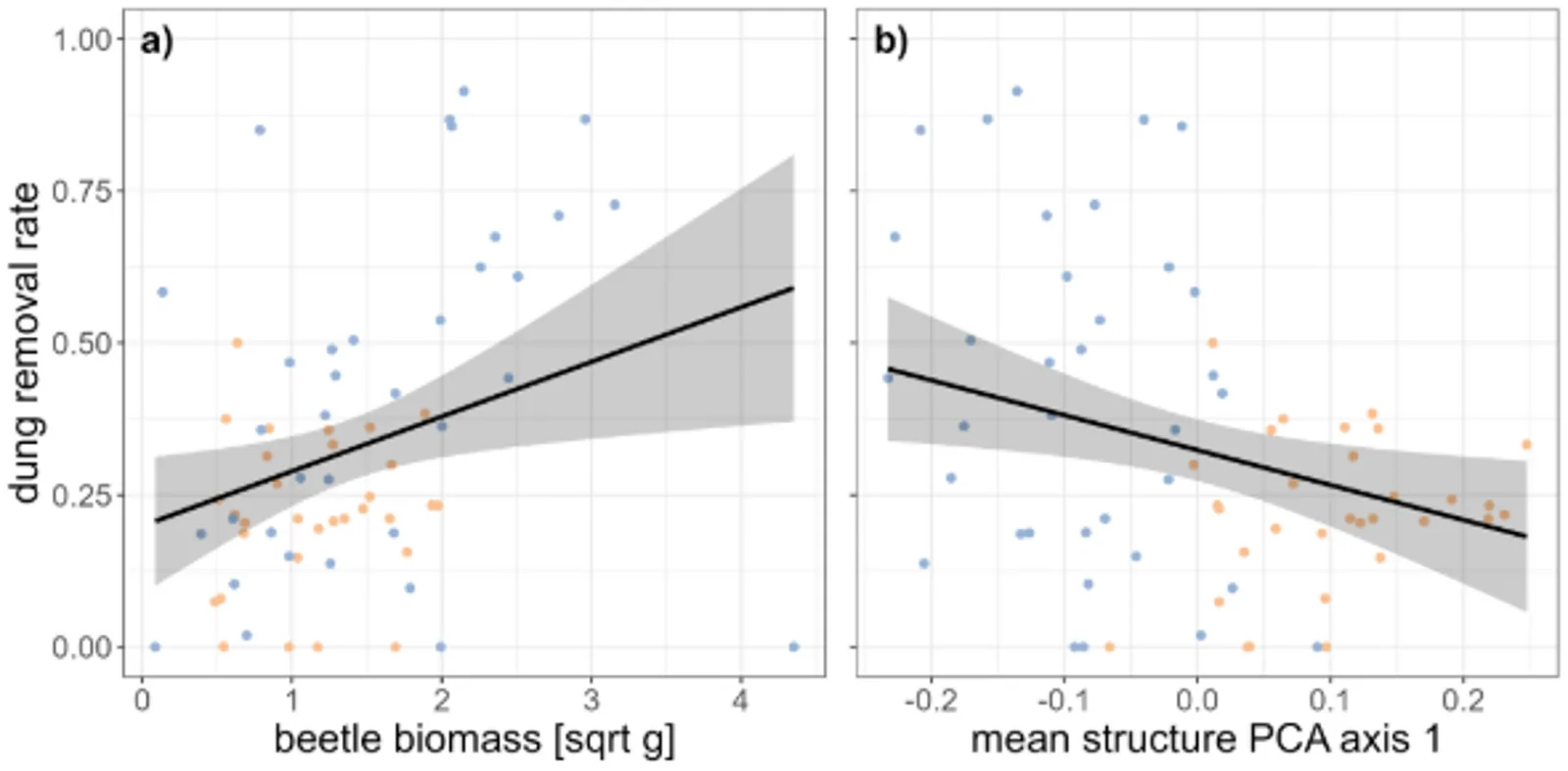
Effects of total dry dung beetle biomass [g] (sqrt-transformed) (**a**) and mean forest structure PCA axis 1 (**b**) on dung removal. The solid lines represent significant effects. Light grey colours indicate the 95% confidence interval. The coloured points represent the two different study areas (blue = eastern forest, orange = western forest). The full model results are given in Table S9

Dung removal increased with higher dung beetle biomass, but was lower in forests characterised by a high proportion of non-natural trees and high herb cover on the forest ground compared to forests characterised by tall and native trees, a large tree basal area and a high canopy cover (mean forest structure PCA axis 1; Fig. 5, Table S9). Additionally, the same statistical effects were found when only *A. stercorosus* biomass was accounted for, while *T. vernalis* biomass alone did not correspond to dung removal (Table S9). Changes in dung beetle diversity and community composition did not correlate with dung removal rates (Table S9-S10).

## Discussion

This study provides insights into the response of dung beetles and dung removal to changes in forest characteristics, which are altered by past forest management and current tree mortality. Our results indicate that the biomass, diversity and community composition of dung beetles are related to changes in mean forest structure. The species *Anoplotrupes stercorosus* (which had the highest biomass) and *Volinus sticticus* (which had the highest abundance) decisively determine biomass and diversity. Furthermore, shifts in the heterogeneity of canopy opening and ground cover, as well as variations in tree diversity, are linked to changes in dung beetle biomass and community composition. Individual dung beetle species responded differently to changes in mean forest structure, while changes in forest structural heterogeneity had no effect. Dung removal rates were higher in forests with high dung beetle biomass, driven by the high biomass of *A. stercorosus*, and in forests with taller trees, a higher proportion of native trees and a larger tree basal area.

The data analysed in this study was limited to forest structural characteristics and forest ground coverage, neglecting the potential influence of soil conditions on dung beetle communities and dung removal (von Hoermann et al. 2020). Dung beetles, such as *A. stercorosus* and *Trypocopris vernalis*, seem to occur in larger quantities on sandy soils (von Hoermann et al. 2020), whereas dung beetle diversity and dung removal could not be affected by the soil type (Asch et al. 2026). As the mean forest structural PCA axes may also reflect changes in soil type, some responses of dung beetle communities and dung removal along these axes could be partly associated with soil conditions. However, we explicitly tested for spatial autocorrelation in the residuals of the models to account for their potential spatial clustering. As most residuals were not spatially autocorrelated, it is unlikely that our results for dung beetles and dung removal are driven by spatial pseudoreplication within the eastern loamy sites or the western sandy sites.

### Effects on dung beetle biomass, diversity and community composition

In forests with a more open canopy, a lower tree basal area, a higher proportion of non-natural trees and a thinner litter layer, dung beetle biomass is lower compared to other forests in the study region. Additionally, our results show that a decrease in dung beetle biomass is associated with greater heterogeneity in canopy openness and forest ground cover, and lower tree diversity. Other studies have also found that recent forests and more open habitats have lower dung beetle biomass (Buse and Entling 2020; Frank et al. 2017; Hülsmann et al. 2020). As dung beetles are not directly dependent on trees, we expected only indirect effects of the proportion of non-natural trees on dung beetles (e.g. through changes in canopy openness). In temperate European forests, dung beetle biomass is mostly driven by the forest specialist *A. stercorosus* (Frank et al. 2017; Hülsmann et al. 2020). In this study, the biomass of *A. stercorosus* varied considerably between plots, but accounted for the largest proportion of overall dung beetle biomass. Therefore, *A. stercorosus* can be seen as a keystone species for dung removal. An important factor possibly driving the avoidance of more open and younger forests by dung beetles is predator avoidance (Goßmann et al. 2023). Using various techniques to prey on dung beetles in and on animal faeces, birds are probably the most efficient predators on dung beetles (Young 2015), and their efficiency may even increase in disturbed and more open forests. As predation risk increases with body size (Nakazawa et al. 2007; Remmel and Tammaru 2009), the large-bodied *A. stercorosus* may avoid more open habitats with smaller trees and thinner litter layers because of the higher risk of predation. It is also possible that factors such as physiological constraints are driving the negative response of dung beetle biomass to younger, more open forests with higher herbaceous cover. In more open habitats with higher sunlight penetration, forest-adapted dung beetles show lower reproductive rates (Vessby 2001) and probably cannot cope with the physiological challenges in such drier and lighter habitats (Nervo et al. 2024; Verdú et al. 2019). However, the fact that we did find some individuals of dung beetle species which prefer open or semi-open habitats (see Table S5) could be an indication that forests with an open canopy can be suitable habitat for those species.

In contrast to the negative effects on overall dung beetle biomass, forests with a more open canopy, a lower tree basal area, a higher proportion of non-natural trees and a thinner litter layer favour a higher diversity of dung beetles in the study region. Open forests are known to have a greater variety of dung beetles because species adapted to open habitats are recruited there (Ambrožová et al. 2022). Such areas may resemble forest edges, which typically have greater taxonomic diversity due to the combination of open and forest habitat communities (Pinksen et al. 2021; Vanneste et al. 2024). The diversity of dung beetles did however not consistently respond to changes in forest structural heterogeneity, although such effects have been shown for the diversity of other organisms such as bryophytes, vascular plants, other arthropods or birds (Heidrich et al. 2020; Hekkala et al. 2023; Uhl et al. 2024).

While changes in forest structural heterogeneity from forests with a diverse tree community, homogeneous forest ground cover and canopy openness to forests with opposite forest conditions did not affect dung beetle diversity, they affected the community composition of dung beetles. In addition, our results show that the dung beetle community composition responds to changes in mean forest structures. These changes range from more closed, native forests with taller trees, a larger tree basal area, to more open forests with lower tree heights, a higher proportion of non-natural trees, and higher herb cover on the forest ground. Changes in community composition were only apparent when calculated based on the biomass of the different species. This suggests that changes in forest structures drive changes in biomass distributions among species, probably through changes in the biomass of the large species *A. stercorosus* and *Trypocopris vernalis*. The occurrence of the species studied individually strongly responds to the mean structure of the forest plots but not to their structural heterogeneity. The occurrence of *T. vernalis* is favoured by forests with lower tree height, lower tree basal area and higher proportion of non-natural tree species, while the same forest characteristics lead to a decrease in the occurrence of *A. stercorosus* (only marginally significant) and *Volinus sticticus*. Our results provide new insights into the three species’ preferences for forest structures, in addition to known positive dominance effects of *A. stercorosus* over *T. vernalis* with increasing forest stand age (Marczak 2013). In line with other studies, the probability of occurrence of the forest specialists *A. stercorosus* and *V. sticticus* decreases with increasing tree defoliation and canopy openness (Frank et al. 2017; Koch 1992; Rössner 2012). This is particularly important given the predicted increase in heat waves, droughts and associated tree mortality (Dai 2013; Senf et al. 2020; Trenberth et al. 2014), as *A. stercorosus* is the most common tunneling dung beetle species in Central Europe (Buse and Entling 2020; Staab et al. 2022). *T. vernalis* responds in the opposite way, showing a higher probability of occurrence on plots with increasing defoliation and canopy openness.

### Effects on dung removal

The dung removal rate negatively correlates with a lower dung beetle biomass. Furthermore, the same effect was observed when accounting only for *A. stercorosus* biomass. This suggests that *A. stercorosus* is a keystone species for dung removal in our study region. Alongside the aforementioned effects on dung beetle biomass, this finding aligns with the results of other studies (Buse and Entling 2020; Staab et al. 2022). With lower tree height, a higher canopy openness, a lower tree basal area and a higher proportion of non-natural tree species one can expect higher temperatures and dung removal rates (Ehbrecht et al. 2019; Nervo et al. 2024). However, we did not observe an increase in dung removal with rising temperatures, instead we observed lower dung removal rates in degraded forests, likely due to lower densities of *A. stercorosus*. This is probably because temperatures near the soil surface on the degraded plots sometimes exceed 40 °C (personal observation), which is the temperature at which *A. stercorosus* experiences complete paralysis (Nervo et al. 2024). Even though the morphologically nearly identical *T. vernalis* has a higher probability of occurrence in the degraded and more open forests it does not occur in the same densities as *A. stercorosus*, probably due to the risk of predation and the ability of *A. stercorosus* to switch to litter as an alternative food source to dung (Byk and Semkiw 2010).

In temperate regions, habitat continuity is important for dung beetles (Hülsmann et al. 2020) and the colonisation of degraded forests by species adapted to open habitats that can subsidise *A. stercorosus* could be hampered by the lack of habitat quantity and continuity in the surrounding area and the patchy and unpredictable availability of dung as a food source (Hanski and Cambefort 1991). Furthermore, large bodied dung beetle species adapted to open habitats, such as *Copris*,* Geotrupes*,* Sisyphus* and *Typhaeus*, are very rare or extinct in the study region due to the use of vermicides or the decline of pasture area (Buse et al. 2015; Lumaret et al. 2012). Lighter and warmer conditions could also explain the negative effects on dung removal in forests with a more open canopy, lower tree height and a higher proportion of non-natural trees. Dung beetles as important dung decomposers prefer a water content of more than 75% in their resource (Holter 2016), but under higher solar radiation the water loss of dung could be too fast and dung beetles do not process the resource after shorter time periods. Dung removal in our study was assessed after a 48 h exposure, making it likely that only Geotrupidae account for dung removal. A longer exposure in future studies might increase dung removal, even on sites without Geotrupidae.

Changes in total dung beetle biomass, diversity, and community composition are related to variations in forest characteristics following forest management actions and partial tree dieback in the study region. Dung beetle responses were more pronounced through shifts in mean forest structure than through small-scale structural heterogeneity. In summary, the lower biomass of dung beetles in younger and degraded forests may be due to a combination of factors, including higher predation risk, reduced continuity and availability of open habitats in the surrounding area, physiological constraints, and the need for faster decomposition due to accelerated water loss in dung. The effects on dung beetle biomass, as well as the effects of dung beetle biomass, were mainly attributed to changes in the biomass of the keystone species *A. stercorosus*. This highlights that changes in forest characteristics, particularly mean forest structures, lead to changes in the dung beetle community and subsequently affect dung decomposition rates. In addition to forest management, climate change will have an increasing impact on forest characteristics. Our results suggest that severe impairment of ecosystem functioning may occur if forest management and tree dieback continue to affect characteristic environmental conditions and forest structure in the coming years.

## Supplementary Information

Below is the link to the electronic supplementary material.

Supplementary Material 1

## Data Availability

All underlying data, derived data, and statistical code pertinent to the results is publicly available in https://zenodo.org/records/17248784.
