# Supplementary material for: Forest structural changes affect dung beetle communities and dung removal rates
Source: Oecologia. 2026 Oct 9;208(11):146. doi: 10.1007/s00442-026-05974-7 (PMC13652778; doi:10.1007/s00442-026-05974-7)
Supplement: Supplementary file 1 [file 442_2026_5974_MOESM1_ESM.docx]

**Supporting Information**

**Forest structural changes affect dung beetle communities and dung removal rates in forests**

Julian Lunow^1,2*^; Katja Wehner^3,4^; Matteo Trevisan^4^; Michael Heethoff^4^; Nico Blüthgen^3a^; Nadja K. Simons^1a^

*Corresponding author: Julius-Maximilians-University Würzburg, Biocenter, Department of Conservation Biology and Forest Ecology, Applied Biodiversity Science, 97074 Würzburg, Germany, julian.lunow@leuphana.de

1: Julius-Maximilians-University Würzburg, Biocenter, Department of Conservation Biology and Forest Ecology, Applied Biodiversity Science, 97074 Würzburg, Germany

2: Leuphana University Lüneburg, Institute of Ecology, 21335 Lüneburg, Germany

3: Technical University Darmstadt, Ecological Networks, 64287 Darmstadt, Germany

4: Technical University Darmstadt, Animal Evolutionary Ecology, 64287 Darmstadt, Germany

 a: both senior-authors contributed equally to this study

**Contents**

Figure S1: Map of the study region and plots………………………………………………..…………3

Figure S2: Correlation matrix of the explanatory variables included in the models…………….……..4

Figure S3: Differences in the explanatory variables between the study regions…………….……...….5

Figure S4: Differences in dung beetle community and dung removal rates between the study regions.6

Figure S5: Scaled model residuals within the study region………………………...…………………..7

Figure S6: Differences in scaled model residuals between regions and years………………………….8

Table S1: Included variables in the mean forest structure and structural variability PCA……………..9

Table S2: Correlation matrix p-values of our included explanatory variable…………………………10

Table S3: Moran*’s* I coefficients and their standard deviation of the dung beetle model residuals…..11

Table S4: Pairwise comparisons of scaled model residuals between the two different regions………12

Table S5: Species list with abundance, biomass and macrohabitat…………………………………...13

Table S6: Effects of the PCAs on dung beetle biomass, Shannon diversity and species richness……14

Table S7: PERMANOVA results on correlation of dung beetles with PCA axes……………...……..15

Table S8: Effects of the PCAs on single species occurrence……………………………….…………16

Table S9: Effects of biomass, Shannon diversity, species richness and PCA axes on dung removal...17

Table S10: PERMANOVA results on correlation of dung beetles and dung removal………………..18

References……………………………………………………………………………………………..18

**Figure S1:** Location of the surveyed plots within the study region in the Darmstadt city forest. The coloured points represent the two different study areas (blue = eastern forest, orange = western forest).

**Figure S2:** Pearson’s r correlation between the explanatory variables mean forest structure PCA axes (mean str. 1 and mean str. 2) and structural heterogeneity PCA axes (str. het. 1 and str. het. 2) used in our models.

**Figure S3:** Differences in the explanatory variables between the two different study regions and soil conditions. The p-values < 0.05, derived from two-tailed t-tests, indicate significant differences in the explanatory variables between the two regions and soil conditions.

**Figure S4:** Differences in dung beetle biomass, Shannon diversity and species richness, and dung removal between the two different study regions and soil conditions. The p-values < 0.05, derived from two-tailed t-tests, indicate significant differences in the explanatory variables between the two regions and soil conditions.

**Figure S5:** Geographical distribution of the scaled model residuals within the study region by the different models (plot header). Red dots indicate residuals below the expectations and blue dots residuals above expectations. Dot size is scaled by the distance to the residual expectation (0-0.5).

**Figure S6:** Scaled model residuals per year and region. Colors indicate eastern (blue) and western (orange) regions. Values > 0.5 are residual values above expectation, while values < 0.5 are residual values below expectations.

**Table S1:** Included variables in the mean forest structure and structural variability PCA. PC1 and PC2 values indicate the correlation of the environmental variables to the two different axes of the different PCAs. R2 is the association of the environmental variable to the ordination and the p-values show if this association is significant. Bold correlations were shown in the graph in the main text.

| PCA | variable | PC1 | PC2 | R2 | p-value |
| --- | --- | --- | --- | --- | --- |
| mean forest | mean DBH | -0.6980 | 0.7161 | 0.8147 | 0.001 |
| structure | mean height | -0.9451 | 0.3268 | 0.8069 | 0.001 |
|  | mean canopy openness | 0.7770 | 0.6296 | 0.6383 | 0.001 |
|  | foliage cover | -0.9936 | -0.1133 | 0.7280 | 0.001 |
|  | moss cover | 0.4007 | -0.9162 | 0.1496 | 0.002 |
|  | bare ground cover | -0.6945 | -0.7195 | 0.0310 | 0.351 |
|  | deadwood cover | -0.8338 | -0.5520 | 0.0116 | 0.676 |
|  | herb cover | 0.9461 | 0.3240 | 0.7818 | 0.001 |
|  | small tree cover | 0.9537 | 0.3008 | 0.0833 | 0.055 |
|  | large tree cover | -0.2139 | -0.9769 | 0.0529 | 0.159 |
|  | basal area | -0.9999 | 0.0167 | 0.5994 | 0.001 |
|  | prop. non-nat. trees | 0.9886 | -0.1506 | 0.2072 | 0.001 |
|  | deadwood volume | 0.7563 | 0.6542 | 0.3395 | 0.001 |
|  | defoliation | 0.3831 | 0.9237 | 0.6819 | 0.001 |
|  | stand age | -0.5370 | 0.8436 | 0.6912 | 0.001 |
|  | foliage height | -0.9987 | 0.0504 | 0.6401 | 0.001 |
| structural | DBH CV | -0.8707 | 0.4917 | 0.8669 | 0.001 |
| heterogeneity | height CV | -0.8776 | 0.4795 | 0.8565 | 0.001 |
|  | CV canopy openness | -0.6243 | 0.7812 | 0.1444 | 0.010 |
|  | gc hetero | -0.6893 | -0.7245 | 0.1353 | 0.005 |
|  | tree richness | -0.6838 | -0.7297 | 0.8351 | 0.001 |
|  | tree diversity | -0.7195 | -0.6945 | 0.7755 | 0.001 |

**Table S2:** Correlation matrix p-values of our included explanatory variable (mean forest structure PCA axis 1, mean forest structure PCA axis 2, structural heterogeneity PCA axis 1, structural heterogeneity PCA axis 1)

| p-values | mean forest structure PCA 1 | mean forest structure PCA 2 | structural heterogeneity PCA 1 | structural heterogeneity PCA 2 |
| --- | --- | --- | --- | --- |
| mean forest structure PCA 1 | 0.00 | 0.36 | 0.99 | 0.34 |
| mean forest structure PCA 2 | 0.36 | 0.00 | 0.64 | 0.68 |
| structural heterogeneity PCA 1 | 0.99 | 0.64 | 0.00 | 0.39 |
| structural heterogeneity PCA 2 | 0.34 | 0.68 | 0.39 | 0.00 |

**Table S3:** Observed and expected Moran’s I coefficients and their standard deviation of the dung beetle model residuals for different years calculated using testSpatialAutocorrelation function of the DHARMa package (Hartig, 2024). P-values < 0.05 indicate spatial autocorrelation of the model residuals. In case of spatial autocorrelation, we fitted a new model with region as additional random effect, indicated by “YES” in the “region incl.” column.

| model | year | region incl. | observed | expected | sd | p-value | spatial autocorrelation |
| --- | --- | --- | --- | --- | --- | --- | --- |
| biomass | 2023 | NO | 0.007 | -0.015 | 0.031 | 0.479 | NO |
| biomass | 2024 | NO | -0.020 | -0.015 | 0.032 | 0.884 | NO |
| diversity | 2023 | NO | 0.032 | -0.015 | 0.031 | 0.135 | NO |
| diversity | 2024 | NO | -0.034 | -0.015 | 0.032 | 0.559 | NO |
| species richness | 2023 | NO | 0.129 | -0.015 | 0.031 | <0.001 | YES |
| species richness | 2024 | NO | -0.0002 | -0.015 | 0.032 | 0.631 | NO |
| species richness | 2023 | YES | 0.242 | -0.015 | 0.031 | <0.001 | YES |
| species richness | 2024 | YES | -0.004 | -0.015 | 0.032 | 0.708 | NO |
| A. stercorosus | 2023 | NO | 0.079 | -0.015 | 0.031 | 0.003 | YES |
| A. stercorosus | 2024 | NO | -0.041 | -0.015 | 0.032 | 0.414 | NO |
| A. stercorosus | 2023 | YES | -0.028 | -0.015 | 0.031 | 0.692 | NO |
| A. stercorosus | 2024 | YES | 0.011 | -0.015 | 0.032 | 0.411 | NO |
| V. sticticus | 2023 | NO | 0.045 | -0.015 | 0.031 | 0.055 | NO |
| V. sticticus | 2024 | NO | -0.026 | -0.015 | 0.032 | 0.744 | NO |
| T. vernalis | 2023 | NO | -0.002 | -0.015 | 0.031 | 0.684 | NO |
| T. vernalis | 2024 | NO | 0.000 | -0.015 | 0.032 | 0.631 | NO |
| dung removal | 2024 | NO | 0.006 | -0.015 | 0.032 | 0.503 | NO |
| dung removal (A. ster. BM) | 2024 | NO | 0.001 | -0.015 | 0.032 | 0.610 | NO |
| dung removal (T. vern. BM) | 2024 | NO | -0.002 | -0.015 | 0.032 | 0.676 | NO |

**Table S4:** ANOVA or Wilcoxon test results for pairwise comparisons of scaled model residuals between the two different regions (East and West). P-values < 0.05 indicate significant differences in the model residuals between the two regions. In case of spatial autocorrelation (Table S3), we fitted a new model with region as additional random effect, indicated by “YES” in the “region incl.” column.

| model | year | region incl. | test | p-value | significant |
| --- | --- | --- | --- | --- | --- |
| biomass | 23 | NO | ANOVA | 0.829 | NO |
| biomass | 24 | NO | Wilcox test | 0.792 | NO |
| diversity | 23 | NO | ANOVA | 0.762 | NO |
| diversity | 24 | NO | ANOVA | 0.754 | NO |
| species richness | 23 | NO | ANOVA | 0.412 | NO |
| species richness | 24 | NO | ANOVA | 0.754 | NO |
| A. stercorosus old | 23 | NO | ANOVA | 0.020 | YES |
| A. stercorosus old | 24 | NO | Wilcox test | 0.451 | NO |
| A. stercorosus | 23 | YES | ANOVA | 0.183 | NO |
| A. stercorosus | 24 | YES | ANOVA | 0.297 | NO |
| V. sticticus | 23 | NO | ANOVA | 0.034 | YES |
| V. sticticus | 24 | NO | ANOVA | 0.593 | NO |
| T. vernalis | 23 | NO | ANOVA | 0.343 | NO |
| T. vernalis | 24 | NO | ANOVA | 0.149 | NO |
| dung removal | 24 | NO | Wilcox test | 0.492 | NO |
| dung removal (A. ster. BM) | 24 | NO | Wilcox test | 0.616 | NO |
| dung removal (T. vern. BM) | 24 | NO | Wilcox test | 0.625 | NO |

**Table S5:** The abundance and biomass of the surveyed species in our study region. The macrohabitat is the preferred habitat of the species according to Buse et al. (2018); Koch, (1992); Rössner (2012).

| species | abundance | biomass [g] | macrohabitat |
| --- | --- | --- | --- |
| *Acrossus depressus*  (Kugelann, 1792) | 109 | 0.643 | semi-open and forest habitat |
| *Acrossus rufipes*  (Linnaeus, 1758) | 2 | 0.081 | semi-open and forest habitat |
| *Anoplotrupes stercorosus*  (Scriba, 1791) | 2,032 | 262.433 | semi-open and forest habitat |
| *Bodilopsis rufa*  (Moll, 1782) | 1 | 0.007 | open and semi-open habitat |
| *Esymus pusillus*  (Herbst, 1789) | 10 | 0.039 | open and semi-open habitat |
| *Euorodalus coenosus*  (Panzer, 1798) | 68 | 0.201 | open and semi-open habitat |
| *Limarus zenkeri*  (Germar, 1813) | 16 | 0.047 | semi-open and forest habitat |
| *Melinopterus prodromus*  (Brahm, 1790) | 5 | 0.027 | open and semi-open habitat |
| *Onthophagus coenobita*  (Herbst, 1783) | 1,112 | 24.030 | semi-open habitat |
| *Onthophagus ovatus*  (Linnaeus, 1767) | 77 | 0.217 | open and semi-open habitat |
| *Onthophagus similis*  (Scriba, 1790) | 2 | 0.022 | semi-open habitat |
| *Onthophagus verticicornis*  (Laicharting, 1781) | 392 | 10.898 | semi-open and forest habitat |
| *Trypocopris vernalis*  (Linnaeus, 1758) | 204 | 47.153 | open and semi-open habitat |
| *Typhaeus typhoeus*  (Linnaeus, 1758) | 1 | 0.144 | semi-open habitat |
| *Volinus sticticus*  (Panzer, 1798) | 6,712 | 24.969 | semi-open and forest habitat |
|  |  |  |  |
| **Total** | **10,743** | **370.909** |  |

**Table S6:** Summary of the effects of the PCAs on biomass (sqrt-transformed), Shannon diversity (e^H^) and species richness. Significant values are in bold. R2m is the marginal explained variance by the overall model.

| response | predictor | estimate | std. error | z-value | p-value | sign. | R2m |
| --- | --- | --- | --- | --- | --- | --- | --- |
| biomass | **str. PCAax1** | **-2.3921** | **0.4951** | **-4.8311** | **< 0.001** | *** | 0.22 |
| (sqrt-trans.) | str. PCAax2 | -0.3248 | 0.5188 | -0.6261 | 0.531 |  |  |
|  | het. PCAax1 | -0.5954 | 0.5027 | -1.1844 | 0.236 |  |  |
|  | **het. PCAax2** | **-1.4723** | **0.5321** | **-2.7671** | **0.006** | ** |  |
| Shannon | **str. PCAax1** | **2.1656** | **0.6511** | **3.3263** | **< 0.001** | *** | 0.09 |
| diversity | str. PCAax2 | -0.4847 | 0.6823 | -0.7105 | 0.477 |  |  |
|  | het. PCAax1 | -0.2791 | 0.6612 | -0.4221 | 0.67 |  |  |
|  | het. PCAax2 | -0.5982 | 0.6996 | -0.8550 | 0.393 |  |  |
| Species | str. PCAax1 | -1.416 | 1.0007 | -1.415 | 0.157 |  | 0.07 |
| richness | str. PCAax2 | -1.248 | 1.059 | -1.178 | 0.239 |  |  |
|  | het. PCAax1 | -1.073 | 0.9855 | -1.089 | 0.276 |  |  |
|  | het. PCAax2 | -1.65 | 1.0327 | -1.597 | 0.11 |  |  |

**Table S7:** Results of the PERMANOVA on the correlation of dung beetle (db) community distances and the forest structure and forest heterogeneity. Reported are response variables, predictor variables, degrees of freedom (Df), Sum of squares (SumOfSqs), R², F-values and p-values.

| response | predictor | Df | SumOfSqs | R2 | F | p value | Sign. |
| --- | --- | --- | --- | --- | --- | --- | --- |
| db community | str. PCAax1 | 1 | 0.215 | 0.006 | 0.861 | 0.456 |  |
| (Bray-Curtis; | str. PCAax2 | 1 | 0.401 | 0.012 | 1.604 | 0.185 |  |
| abundance | het. PCAax1 | 1 | 0.159 | 0.005 | 0.636 | 0.633 |  |
| based) | het. PCAax2 | 1 | 0.375 | 0.011 | 1.500 | 0.200 |  |
|  | Residual | 128 | 31.961 | 0.965 |  |  |  |
|  | Total | 132 | 33.110 | 1 |  |  |  |
| db community | **str. PCAax1** | **1** | **0.663** | **0.028** | **3.850** | **0.009** | ** |
| (Bray-Curtis; | str. PCAax2 | 1 | 0.094 | 0.004 | 0.545 | 0.695 |  |
| biomass | het. PCAax1 | 1 | 0.342 | 0.014 | 1.985 | 0.124 |  |
| based) | **het. PCAax2** | **1** | **0.531** | **0.022** | **3.085** | **0.037** | * |
|  | Residual | 128 | 22.036 | 0.931 |  |  |  |
|  | Total | 132 | 23.665 | 1 |  |  |  |
| db community | str. PCAax1 | 1 | 0.226 | 0.005 | 0.729 | 0.613 |  |
| (Jaccard) | str. PCAax2 | 1 | 0.421 | 0.010 | 1.359 | 0.220 |  |
|  | het. PCAax1 | 1 | 0.255 | 0.006 | 0.824 | 0.557 |  |
|  | het. PCAax2 | 1 | 0.536 | 0.013 | 1.732 | 0.112 |  |
|  | Residual | 128 | 39.615 | 0.965 |  |  |  |
|  | Total | 132 | 41.051 | 1 |  |  |  |

**Table S8:** Summary of the effects of the PCAs on single species occurrence of Anoplotrupes stercorosus, Trypocopris vernalis and Volinus sticticus. Significant values are bold. R2m is the marginal explained variance by the overall model.

| response | predictor | estimate | std. error | z-value | p-value | sign. | R2m |
| --- | --- | --- | --- | --- | --- | --- | --- |
| *A. stercorosus* | str. PCAax1 | -5.631 | 2.875 | -1.958 | 0.050 | . | 0.25 |
|  | **str. PCAax2** | **-6.566** | **2.888** | **-2.273** | **0.023** | * |  |
|  | het. PCAax1 | -1.308 | 2.367 | -0.553 | 0.581 |  |  |
|  | het. PCAax2 | -0.443 | 2.647 | -0.168 | 0.867 |  |  |
| *T. vernalis* | **str. PCAax1** | **12.414** | **2.301** | **-5.822** | **< 0.001** | *** | 0.62 |
|  | **str. PCAax2** | **5.536** | **2.069** | **-4.243** | **0.007** | ** |  |
|  | het. PCAax1 | -1.966 | 1.839 | 0.553 | 0.285 |  |  |
|  | het. PCAax2 | -1.053 | 2.160 | 0.943 | 0.626 |  |  |
| *V. sticticus* | **str. PCAax1** | **-16.122** | **2.769** | **5.394** | **< 0.001** | ******* | 0.44 |
|  | **str. PCAax2** | **-11.108** | **2.618** | **2.676** | **< 0.001** | ******* |  |
|  | het. PCAax1 | 1.128 | 2.038 | -1.069 | 0.580 |  |  |
|  | het. PCAax2 | 2.348 | 2.489 | -0.488 | 0.345 |  |  |

**Table S9:** Summary of the effects of biomass (overall, A. stercorosus (AS) and T. vernalis (TV); sqrt-transformed), Shannon diversity (e^H^), species richness and the environmental PCA axes on dung removal. Significant values are bold. R2m is the marginal explained variance by the overall model.

| response | predictor | estimate | std. error | z-value | p-value | sign. | R2m |
| --- | --- | --- | --- | --- | --- | --- | --- |
| dung removal | **biomass (sqrt)** | **0.134** | **0.044** | **3.059** | **0.002** | * | 0.27 |
|  | shannon diversity | 0.055 | 0.047 | 1.154 | 0.248 |  |  |
|  | species richness | -0.064 | 0.036 | -1.755 | 0.079 |  |  |
|  | **str. PCAax1** | **-0.602** | **0.230** | **-2.619** | **0.009** | * |  |
|  | str. PCAax2 | -0.298 | 0.222 | -1.341 | 0.180 |  |  |
|  | het. PCAax1 | 0.107 | 0.219 | 0.489 | 0.625 |  |  |
|  | het. PCAax2 | 0.102 | 0.238 | 0.427 | 0.669 |  |  |
| dung removal | **biomass AS (sqrt)** | **0.102** | **0.041** | **2.453** | **0.014** | * | 0.24 |
|  | shannon diversity | 0.03 | 0.047 | 0.644 | 0.519 |  |  |
|  | species richness | -0.041 | 0.035 | -1.179 | 0.238 |  |  |
|  | str. PCAax1 | -0.447 | 0.264 | -1.691 | 0.091 |  |  |
|  | str. PCAax2 | -0.152 | 0.23 | -0.660 | 0.509 |  |  |
|  | het. PCAax1 | 0.12 | 0.224 | 0.533 | 0.594 |  |  |
|  | het. PCAax2 | 0.081 | 0.246 | 0.332 | 0.74 |  |  |
| dung removal | biomass TV (sqrt) | 0.062 | 0.079 | 0.784 | 0.433 |  | 0.17 |
|  | shannon diversity | -0.0002 | 0.047 | -0.003 | 0.997 |  |  |
|  | species richness | -0.005 | 0.032 | -0.149 | 0.881 |  |  |
|  | **str. PCAax1** | **-0.927** | **0.305** | **-3.04** | **0.002** | ** |  |
|  | str. PCAax2 | -0.352 | 0.27 | -1.303 | 0.193 |  |  |
|  | het. PCAax1 | 0.173 | 0.233 | 0.741 | 0.459 |  |  |
|  | het. PCAax2 | -0.087 | 0.245 | -0.354 | 0.723 |  |  |

**Table S10:** Results of the PERMANOVA on the correlation of dung beetle (db) community distances and dung removal. Reported are response variables, predictor variables, degrees of freedom (Df), Sum of squares (SumOfSqs), R², F-values and p-values.

| response | predictor | Df | SumOfSqs | R2 | F-value | p-value |
| --- | --- | --- | --- | --- | --- | --- |
| db community | dung removal | 1 | 0.295 | 0.014 | 0.927 | 0.460 |
| (Bray-Curtis; | Residual | 64 | 20.384 | 0.986 |  |  |
| abundance based) | Total | 65 | 20.680 | 1.000 |  |  |
| db community | dung removal | 1 | 0.201 | 0.011 | 0.686 | 0.632 |
| (Bray-Curtis; | Residual | 64 | 18.797 | 0.989 |  |  |
| biomass based) | Total | 65 | 18.998 | 1.000 |  |  |
| db community | dung removal | 1 | 0.202 | 0.011 | 0.686 | 0.63 |
| (Jaccard) | Residual | 64 | 18.797 | 0.989 |  |  |
|  | Total | 65 | 18.998 | 1.00 |  |  |

**References**

Buse, J., Šlachta, M., Sladecek, F.X.J., Carpaneto, G.M., 2018. Summary of the morphological and ecological traits of Central European dung beetles. Entomol. Sci. 21, 315–323. https://doi.org/10.1111/ens.12313

Hartig, F., 2024. DHARMa: Residual diagnostics for hierarchical (multi-Level / mixed) regression models.

Koch, K., 1992. Die Kaefer Mitteleuropas. Oekologie. Goecke & Evers, Krefeld.

Rössner, E., 2012. Die Hirschkäfer und Blatthornkäfer Ostdeutschlands (Coleoptera: Scarabaeoidea). Verein der Freunde und Förderer des Naturkundemuseums Erfurt, Erfurt.
